# Genome-wide analysis of the rice phloem protein 2 gene family identifies *OsPP2–10* and *OsPP2–13* as candidate genes associated with salt and osmotic stress responses

**DOI:** 10.3389/fpls.2026.1953442

**Published:** 2026-10-21

**Authors:** Yongdong Dai, Yang Chen

**Affiliations:** 1 Biotechnology Research Institute, Chongqing Academy of Agricultural Sciences, Chongqing, China; 2 Chongqing Key Laboratory of Adversity Agriculture Research, Chongqing, China; 3 Key Laboratory of Evaluation and Utilization for Special Crops Germplasm Resource in the Southwest Mountains, Ministry of Agriculture and Rural Affairs, Chongqing, China

**Keywords:** abiotic stress, genome-wide analysis, oryza sativa, phloem protein (PP2), phylogenetic analysis

## Abstract

**Introduction:**

Phloem protein 2 (PP2) proteins are major structural components of the phloem and play important roles in long-distance signaling and plant responses to environmental stresses. However, the evolutionary history and potential functions of the PP2 gene family in rice remain poorly understood.

**Methods:**

We identified 20 OsPP2 genes and analyzed their evolutionary relationships, gene structures, duplication events, promoter cis-regulatory elements, and expression profiles. Quantitative real-time PCR (qRT-PCR) was used to validate stress-responsive expression patterns. Subcellular localization and yeast heterologous expression assays were performed to further investigate the potential functions of *OsPP2-10* and *OsPP2-13*.

**Results:**

Both segmental and tandem duplication contributed to the expansion of the OsPP2 family. Promoter analysis identified multiple predicted stress- and hormone-responsive cis-regulatory elements in most OsPP2 genes. Expression profiling revealed distinct tissue-specific patterns and differential responses to salt, drought, heat, and cold stresses. *OsPP2-10* and *OsPP2-13* showed relatively strong expression responses to salt stress and PEG6000-induced osmotic stress, consistent with the qRT-PCR results. Subcellular localization analysis detected both proteins in the nucleus and peripheral cellular regions. Heterologous expression of these genes enhanced yeast tolerance to salt and osmotic stress.

**Discussion:**

These findings provide a systematic overview of the rice PP2 gene family and preliminary evidence supporting the potential involvement of *OsPP2-10* and *OsPP2-13* in abiotic stress responses. Both genes represent promising candidates for further functional investigation in rice.

## 1 Introduction

Rice (*Oryza sativa* L.) is one of the world’s most important staple crops, providing food for more than half of the global population. Ensuring stable rice production and improving yield are therefore essential for global food security (Fan et al., 2023). However, climate change is increasing the frequency and severity of abiotic stresses, among which drought and soil salinity are major constraints on crop growth and productivity (Zhang et al., 2022). Salt and drought stresses impose complex physiological constraints on plants, including osmotic imbalance, ion toxicity, and oxidative damage, and activate partially overlapping signaling networks involving phytohormones, reactive oxygen species (ROS), and transcriptional reprogramming (He et al., 2024; Liu et al., 2024a). Recent studies in crops have further shown that ROS homeostasis, antioxidant capacity, and ion balance are closely associated with salt stress tolerance (Li et al., 2025; Xiao et al., 2026). Therefore, understanding the molecular components that coordinate these physiological responses is important for elucidating the mechanisms of stress adaptation in rice.

In plants, the vascular system serves as a major route for long-distance communication between different organs. In addition to transporting photo assimilates, the phloem carries proteins, RNAs, phytohormones, and other mobile signals that coordinate developmental processes and systemic responses to environmental stimuli (Koenig and Hoffmann-Benning, 2020; Li et al., 2026). This function is particularly relevant to abiotic stress responses, because stress signals perceived locally in roots or shoots must be transmitted and integrated across the plant to coordinate physiological adaptation in distal tissues. Thus, phloem-associated proteins are likely to have functions extending beyond transport and wound responses and may participate in the regulation of systemic stress signaling.

Phloem Protein 2 (PP2), originally identified in plant phloem sap, comprises a class of lectin-like proteins that are widely distributed in the phloem of higher plants (Dinant et al., 2003). PP2 proteins have traditionally been linked to phloem sealing and protection after wounding, but accumulating evidence indicates that they also contribute to plant defense responses. For example, the *Arabidopsis thaliana* protein AtPP2-A1 exhibits both molecular chaperone and antifungal activities and contributes to defense against pathogen infection and insect attack (Du et al., 2022a; Lee et al., 2014; Zhang et al., 2011). *PP2-A5* enhances resistance to *Tetranychus urticae* by modulating phytohormone signaling (Santamaría et al., 2019). In citrus, overexpression of PP2 genes enhances phloem callose deposition, significantly reduces the abundance of *Candidatus Liberibacter asiaticus* (CLas), and suppresses disease development (Dai et al., 2026; Zeng et al., 2022). These findings indicate that PP2 proteins are involved in processes closely associated with phloem integrity and defense, providing a basis for investigating their potential roles in broader stress responses.

Beyond their established roles in phloem-associated defense, increasing evidence has linked PP2 proteins to abiotic stress responses. Studies have shown that the *Arabidopsis* SCF E3 ubiquitin ligase gene *AtPP2-B11* positively regulates salt tolerance by modulating ROS accumulation and Na^+^ homeostasis, while negatively affecting drought tolerance (Jia et al., 2015; Li et al., 2014). Another *Arabidopsis* PP2-encoding gene, *AFA1*, negatively regulates ABA signaling under stress conditions (Kim et al., 2021). In cucumber, overexpression of *CsPP2-A1* enhances both salt tolerance and resistance to aphid infestation (Du et al., 2022b; Li et al., 2023). Silencing *GhPP2–33* reduces salt tolerance in cotton, and ThNAC13 enhances salt tolerance in *Tamarix* by activating *ThPP2* expression, reducing ROS accumulation, and increasing antioxidant enzyme activity (Liu et al., 2024b; Wei et al., 2023). Collectively, these findings suggest that, in addition to their established roles in phloem-associated defense, individual PP2 proteins may contribute to the regulation of abiotic stress responses through pathways associated with ABA signaling, ROS homeostasis, and ion balance.

With the rapid development of plant genomics and bioinformatics, genome-wide analyses of the PP2 gene family have been carried out in multiple plant species. For example, a recent genome-wide study identified 15 PP2 genes in cassava, with several members showing distinct expression patterns across tissues and selected PP2 genes responding to drought and cold stresses (Aluko et al., 2026). However, comprehensive characterization of the rice PP2 gene family remains limited. To date, only a few studies have suggested that *OsFBX148* functions as a component of the SCF complex and may regulate ABA signaling and ROS accumulation. Moreover, overexpression of *OsFBX148* in *Arabidopsis* enhances salt tolerance (Fan et al., 2021; Wang et al., 2024). Although these findings suggest that PP2 genes play important roles in rice stress responses, a comprehensive characterization of the rice PP2 gene family is still lacking, and its expression patterns and functions under abiotic stresses remain largely unknown.

Therefore, we performed a genome-wide identification and comprehensive analysis of the rice PP2 gene family to investigate its evolutionary characteristics, expression patterns, and potential roles in abiotic stress responses. Our findings provide a useful resource for future functional studies and for exploring candidate PP2 genes involved in stress responses in rice.

## 2 Materials and methods

### 2.1 Identification of PP2 family members in rice

The genome sequence and corresponding annotation files of rice (*Oryza sativa* L. ssp. *japonica* cv. Nipponbare, version AGIS-1.0) were downloaded from the RiceSuperPIR database (http://www.ricesuperpir.com/) (Shang et al., 2023). When multiple transcript isoforms were annotated for the same gene locus, the longest annotated isoform was retained. To identify candidate PP2 family members, the hidden Markov model (HMM) profile of the PP2 conserved domain (PF14299) was downloaded from the Pfam database and used to search the rice proteome using the HMM Search function in TBtools, with an E-value cutoff of 1e-5 (Chen et al., 2023). In parallel, previously characterized PP2 protein sequences from *Arabidopsis thaliana* were used as queries for BLASTP searches against the rice proteome using an E-value cutoff of 1e-5 to avoid overlooking potential family members. All nonredundant candidate proteins were subsequently examined using the Conserved Domain Database (https://www.ncbi.nlm.nih.gov/Structure/cdd/wrpsb.cgi) and InterPro (https://www.ebi.ac.uk/interpro/) to confirm the presence of the PP2 domain (Letunic et al., 2021; Lu et al., 2020). Proteins were retained as OsPP2 candidates when a complete PP2 domain corresponding to PF14299 was detected by domain annotation and showed no obvious truncation. The confirmed OsPP2 genes were named according to their chromosomal locations. The correspondence among the study-specific OsPP2 identifiers, official rice locus IDs, and, where available, previously reported gene names is provided in **Supplementary Table S4**. The physicochemical properties of the identified OsPP2 proteins were predicted using the Protein Parameter Calculator module in TBtools.

### 2.2 Chromosomal distribution and phylogenetic analysis

The chromosomal locations of OsPP2 genes were extracted from the genome annotation file and visualized using TBtools. Gene density along the chromosomes was calculated using a 200-kb sliding window. To investigate the evolutionary relationships of the PP2 family, full-length PP2 protein sequences from rice, *Arabidopsis thaliana*, and *Citrus sinensis* were retrieved from public databases. Multiple sequence alignment was performed using the ClustalW algorithm implemented in MEGA X software (Kumar et al., 2018). A phylogenetic tree was constructed using the maximum-likelihood method with 1,000 bootstrap replicates. The resulting phylogenetic tree was visualized and annotated using the iTOL online platform (https://itol.embl.de/) (Letunic and Bork, 2024). Bootstrap values were displayed at the corresponding nodes.

### 2.3 Gene structure and conserved motif analysis

The exon–intron structures of OsPP2 genes were analyzed based on the rice genome annotation file and visualized using TBtools. Conserved motifs in OsPP2 proteins were identified using the MEME online program (https://meme-suite.org/meme/tools/meme), with the maximum number of motifs set to six, while all other parameters were retained at their default settings (Bailey et al., 2009). The distributions of conserved motifs and gene structures were subsequently integrated and visualized using TBtools.

### 2.4 Gene duplication and synteny analysis

To investigate the evolutionary expansion patterns of the PP2 gene family, intraspecies synteny analysis within the rice genome was performed using the MCScanX program. The parameters used for MCScanX analysis were as follows: a minimum of 5 genes per synteny block, an E-value cutoff of 1 × 10^−5^, a gap penalty of −1, a maximum gap of 20, and a match score of 50. The resulting syntenic relationships were used to identify duplicated OsPP2 gene pairs. Duplication modes were further classified using the MCScanX duplicate gene classification procedure. For interspecies synteny analysis, orthologous relationships between rice PP2 genes and those of *Arabidopsis thaliana*, *Glycine max*, *Zea mays*, and *Sorghum bicolor* were identified using MCScanX implemented in TBtools. Syntenic maps were generated to illustrate orthologous gene pairs among different species. The nonsynonymous substitution rate (Ka), synonymous substitution rate (Ks), and Ka/Ks ratios of duplicated gene pairs were calculated using the KaKs Calculator module in TBtools.

### 2.5 *Cis*-acting element analysis in promoter regions

The 2-kb sequences upstream of the ATG of each OsPP2 gene were extracted as putative promoter regions using TBtools. These promoter sequences were submitted to the PlantCARE database (https://bioinformatics.psb.ugent.be/webtools/plantcare/html/) to predict *cis*-acting regulatory elements (Lescot et al., 2002). Each motif occurrence reported by PlantCARE was counted as one predicted site. When multiple motif types were reported at overlapping or identical positions, they were retained as separate predicted motif types because they represent distinct database annotations. No additional statistical enrichment analysis was performed against a background of genomic promoters; therefore, the occurrence and abundance of *c*is-acting elements were interpreted as descriptive features of the OsPP2 promoter regions. The identified *cis*-acting elements were categorized and visualized using TBtools.

### 2.6 Expression profile analysis of OsPP2 genes in different tissues

To investigate the tissue-specific expression patterns of OsPP2 genes, publicly available transcriptomic datasets were retrieved from the Rice Expression Database (https://ngdc.cncb.ac.cn/red/indexProject), project DRP000391. Expression data from roots (7 days after germination), shoots (7 days after germination), leaves (7 days before heading), panicles (7 days before heading), and seeds of Nipponbare were retrieved and transformed using log_2_(FPKM + 1). The pseudocount of 1 was added to avoid undefined values for zero-expression entries. For heatmap visualization, the log2-transformed expression values were further normalized by row-wise Z-score transformation for each gene across the analyzed tissues. Heatmaps were generated using the HeatMap module implemented in TBtools. Original FPKM values used for generating the heatmap are provided in **Supplementary Table S1**.

### 2.7 Expression profile analysis of OsPP2 genes under abiotic stresses

To assess the responses of OsPP2 genes to abiotic stresses, transcriptomic datasets associated with salt, drought, heat, and cold treatments were retrieved from the Rice Genome Annotation Project database (https://rice.uga.edu/expression.shtml). Each treatment condition included three biological replicates, which were retained as separate samples for expression visualization. Detailed information for each dataset, including rice cultivar, developmental stage, stress types, and treatment durations, is listed in **Supplementary Table S2**. The expression values were transformed using log_2_(FPKM + 1) and subsequently normalized by Z-score transformation. Heatmaps were generated using TBtools to visualize the relative expression patterns of OsPP2 genes under different stress conditions. Original FPKM values used are provided in **Supplementary Table S3**.

### 2.8 Plant materials, stress treatments, and qRT-PCR analysis

To investigate the expression patterns of OsPP2 genes under abiotic stress, two-week-old Nipponbare seedlings were subjected to salt and osmotic stress treatments. Seedlings were treated with either 150 mM NaCl or 20% (w/v) PEG6000, while untreated seedlings sampled at 0 h were used as the baseline control. PEG6000 treatment was used to impose osmotic stress and was not intended to directly reproduce field or soil-based drought conditions. Shoot tissues were collected at 0, 3, 6, 12, and 24 h after treatment, immediately frozen in liquid nitrogen, and stored at −80 °C until further analysis.

To evaluate the transcriptional responses of OsPP2 genes to salt and osmotic stresses, total RNA was extracted from the collected samples using the RNAprep Pure Plant Kit (TIANGEN, Beijing, China). RNA quality and integrity were assessed prior to cDNA synthesis. First-strand cDNA was synthesized from 1 μg of total RNA using the FastKing RT Kit (TIANGEN, Beijing, China). Quantitative real-time PCR (qRT-PCR) was performed using FastFire qPCR SYBR Green Master Mix (TIANGEN, Beijing, China) according to the manufacturer’s instructions. *OsACT1* was used as the reference gene for qRT-PCR because previous studies evaluated its expression stability in rice and recommended its use for shoot tissues under salt and drought stress conditions (Liu et al., 2023). Three biological replicates were included for each treatment and sampling time point. Relative gene expression levels were calculated using the 2^−ΔΔCt^ method. Primer sequences are provided in **Supplementary Table S6**.

### 2.9 Yeast heterologous expression and stress tolerance assay

To evaluate the potential roles of *OsPP2–10* and *OsPP2–13* in abiotic stress tolerance, their coding sequences were cloned into the pYES2 vector. The resulting constructs (pYES2-*OsPP2–10* and pYES2-*OsPP2-13*), together with the empty vector pYES2, were individually transformed into the yeast strain INVSc1. Positive transformants were selected on synthetic dropout medium lacking uracil (SD/-Ura). Single colonies were cultured overnight in SD/-Ura liquid medium and then transferred into SG/-Ura medium to induce transgene expression until the cultures reached the exponential growth phase. For the yeast spot assay, cell suspensions were adjusted to the same optical density (OD_600_ = 1.0) and serially diluted (10^-1^, 10^-2^, 10^-3^) with 1× TE buffer. Aliquots (10 μL) of each dilution were spotted onto SG/-Ura agar plates supplemented with either 300 mM NaCl or 10% PEG6000. SG/-Ura plates without stress treatment served as the control. Plates were incubated at 30 °C for 3 days, after which colony growth was photographed. Primer sequences are provided in **Supplementary Table S6**.

### 2.10 Subcellular localization

To determine the subcellular localization of OsPP2–10 and OsPP2-13, their full-length coding sequences without stop codons were amplified and cloned into the pRC5 vector. The resulting recombinant plasmids, together with the empty-vector control, were introduced into *Agrobacterium tumefaciens* and transiently expressed in *Nicotiana benthamiana* leaf epidermal cells via agroinfiltration. mRFP1-mCherry was used as a nuclear marker. Infiltrated plants were maintained at 28 °C under a 12 h light/12 h dark photoperiod for 72 h before imaging. GFP fluorescence signals were visualized using a Zeiss LSM800 confocal laser scanning microscope (Zeiss, Jena, Germany). Primer sequences used for vector construction are provided in **Supplementary Table S6**.

### 2.11 Statistical analysis

Statistical analyses were performed using GraphPad Prism v9.0. For each gene and stress treatment, the expression levels at each time point were compared with the corresponding 0 h control using two-tailed t-tests. A *P* value < 0.05 was considered statistically significant.

## 3 Results

### 3.1 Identification of the PP2 gene family members in rice

A total of 20 PP2 genes were identified in the rice genome and designated *OsPP2–1* to *OsPP2–20* based on their chromosomal locations. Physicochemical analysis revealed considerable variation among the OsPP2 proteins (**Supplementary Table S4**). The OsPP2 proteins ranged from 166 to 1,016 amino acids in length, with predicted molecular weights ranging from 19,287.3 to 115,455.8 Da. The predicted theoretical isoelectric points (pI) ranged from 4.90 to 9.80. Most OsPP2 proteins had acidic pI values, indicating physicochemical diversity within the family. The instability index ranged from 32.68 to 63.21, and most members exhibited values greater than 40, suggesting that they are likely unstable. The aliphatic index ranged from 68.12 to 91.22, whereas the GRAVY value ranged from −0.480 to −0.064. All OsPP2 proteins exhibited negative GRAVY values, indicating that they are generally hydrophilic.

### 3.2 Chromosomal localization and phylogenetic analysis

Chromosomal localization analysis was performed to determine the genomic distribution of the OsPP2 gene family (**Figure 1A**). The 20 OsPP2 genes were unevenly distributed across eight chromosomes (Chr1, Chr2, Chr3, Chr4, Chr8, Chr9, Chr10, and Chr12). Among these chromosomes, Chr2 contained the largest number of OsPP2 genes, with eight members, followed by Chr12 with four members. Chr4 and Chr9 each harbored two OsPP2 genes, whereas Chr1, Chr3, Chr8, and Chr10 each contained a single gene. Notably, several OsPP2 genes were clustered in the distal region of Chr2, suggesting that gene duplication events may have contributed to their expansion.

**Figure 1:**
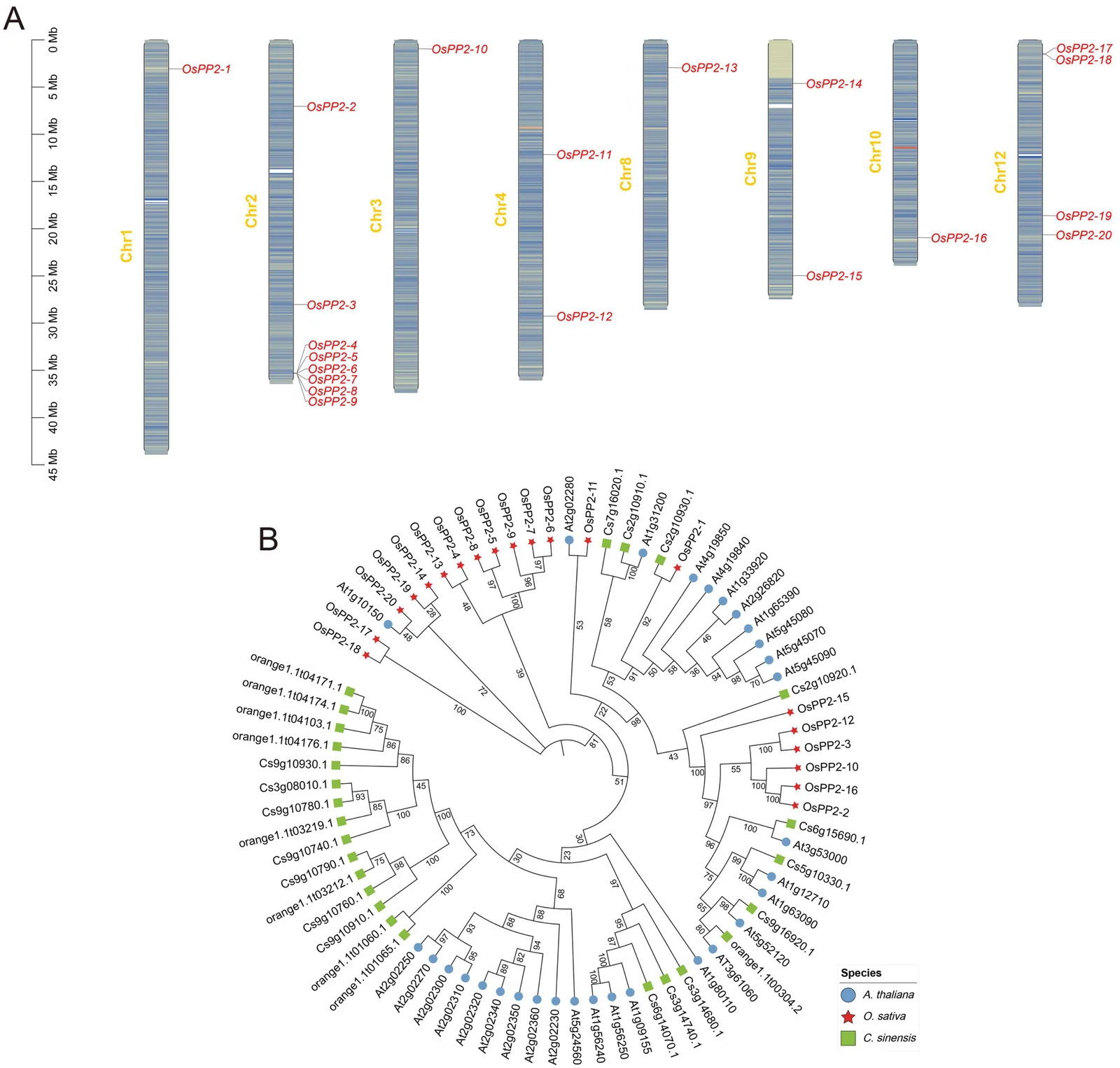
Chromosomal localization and phylogenetic analysis. **(A)** Chromosomal locations of the OsPP2 genes on the rice chromosomes. To assess gene density, each chromosome was analyzed with a 200-kb sliding window, and the resulting densities were represented by a color gradient from blue (low) to red (high). **(B)** Maximum-likelihood phylogenetic tree of PP2 family proteins from *O. sativa*, *A. thaliana* and *C. sinensis*. PP2 proteins from each species are represented by the same symbols. Numbers at the nodes indicate ultrafast bootstrap support values from 1,000 replicates.

A maximum-likelihood phylogenetic tree was constructed using PP2 protein sequences from *Oryza sativa*, *Arabidopsis thaliana*, and *Citrus sinensis* to investigate the evolutionary relationships within the PP2 family (**Figure 1B**). The results indicated that PP2 proteins from the three species could be classified into multiple distinct clades, with proteins from the same species tending to cluster together. These findings suggest that the PP2 gene family has undergone lineage-specific expansion and diversification during plant evolution.

### 3.3 Gene structure and conserved motif analysis

To further characterize the OsPP2 gene family, a phylogenetic tree was constructed using all OsPP2 protein sequences, followed by analyses of gene structure and conserved motifs (**Figure 2A**). Six conserved motifs (Motifs 1–6) were identified (**Figure 2B**; **Supplementary Figure S1**). Motifs 1, 2, 3, and 5 were present in most OsPP2 proteins, indicating that these motifs are highly conserved within the family. The motif composition was largely consistent with the phylogenetic relationships, although differences were observed among individual OsPP2 proteins. For example, *OsPP2–20* exhibited a motif composition markedly different from those of the other members, suggesting structural divergence.

**Figure 2:**
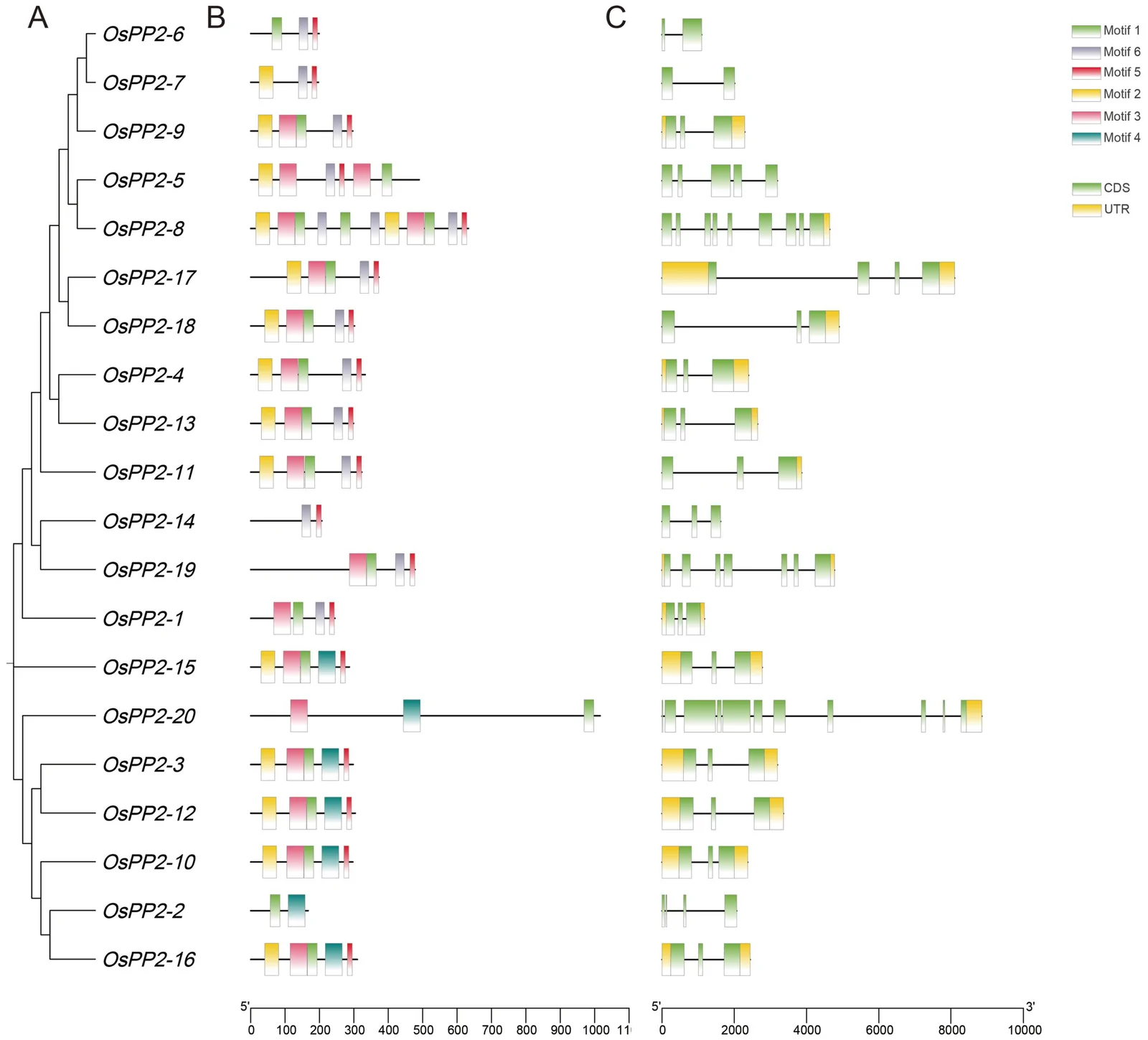
Evolutionary relationships, conserved motifs, and gene structures of the OsPP2 gene family. **(A)** Phylogenetic tree of the OsPP2 family. **(B)** Distribution of conserved motifs in the OsPP2 proteins. Six conserved motifs are indicated by different colors. The scale bar represents the amino acid length of the corresponding OsPP2 proteins. **(C)** Gene structure analysis of OsPP2 genes. The scale bar represents the nucleotide length of the corresponding genes.

Gene structure analysis revealed considerable variation in exon number and intron length among the OsPP2 genes (**Figure 2C**). *OsPP2–20* contained 11 exons, the highest number among the OsPP2 genes, whereas *OsPP2–6* and *OsPP2–7* each contained only two exons, representing the fewest in the family.

### 3.4 Gene duplication and synteny analysis

Gene duplication is a major driving force underlying functional diversification and adaptive evolution (Bowers et al., 2003; Vision et al., 2000). To investigate the evolutionary history of the OsPP2 gene family, both intraspecific and interspecific synteny analyses were performed. In the rice genome, eight duplicated OsPP2 gene pairs were identified based on MCScanX analysis, including six segmentally duplicated pairs and two tandemly duplicated pairs (**Figure 3A**). The two tandemly duplicated pairs, *OsPP2-4*/*OsPP2–5* and *OsPP2-6*/*OsPP2-7*, were located within the OsPP2 gene cluster on chromosome 2, confirming that this region contains local tandem duplication events. Ka/Ks analysis showed that all eight duplicated OsPP2 gene pairs had Ka/Ks ratios below 1, indicating that they have been subjected to purifying selection (**Supplementary Table S5**).

**Figure 3:**
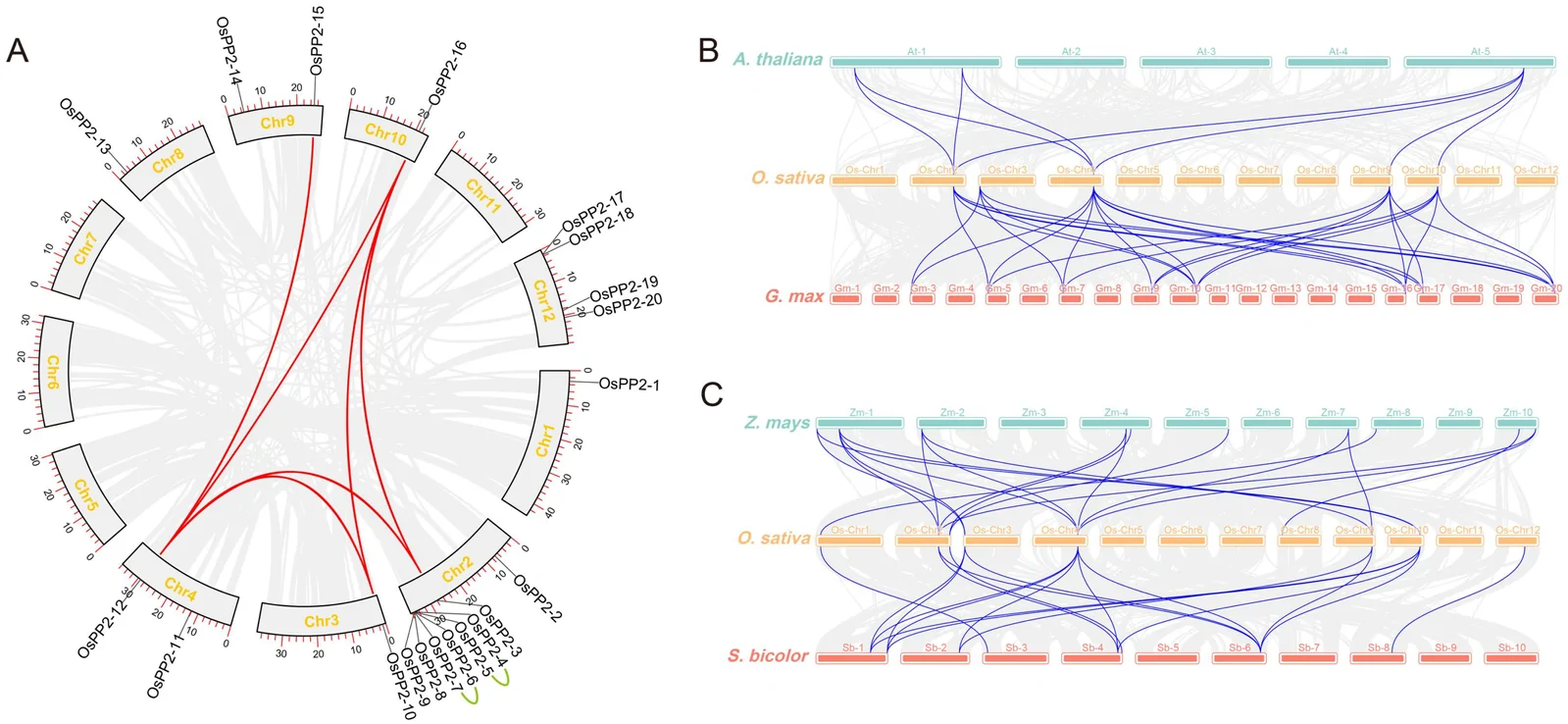
Gene duplication and synteny analysis of OsPP2 genes. **(A)** Intraspecific synteny analysis of OsPP2 genes in the rice genome. Red connecting lines represent segmentally duplicated gene pairs, whereas tandemly duplicated gene pairs are indicated by green lines. Gray lines in the background indicate all syntenic blocks within the rice genome. **(B, C)** Comparative synteny analysis of PP2 genes between *O. sativa* and *A. thaliana* and *G. max* **(B)**, or *Z. mays* and *S. bicolor* **(C)**. Blue lines indicate orthologous syntenic gene pairs.

To further investigate the evolutionary relationships of OsPP2 genes across species, comparative synteny analyses were performed between rice and two monocot species (maize and sorghum) as well as two dicot species (*Arabidopsis* and soybean) (**Figures 3B, C**). The results showed that rice PP2 genes formed 8, 28, 19, and 19 syntenic gene pairs with *Arabidopsis*, soybean, maize, and sorghum, respectively. Notably, rice PP2 genes formed the fewest syntenic gene pairs with *Arabidopsis*, whereas more syntenic pairs were detected with soybean, maize, and sorghum.

### 3.5 *Cis*-acting element analysis

*Cis*-acting elements within promoter regions regulate gene expression in response to developmental cues and environmental signals, thereby contributing to gene regulatory networks (Li and Schmitz, 2025; Marand et al., 2023). To investigate the potential regulatory roles of OsPP2 genes in plant growth, development, and stress responses, the 2-kb sequences upstream of the ATG of all OsPP2 genes were extracted and analyzed for *cis*-acting elements (**Figure 4**). Numerous *cis*-acting elements associated with hormone responsiveness, abiotic stress responses, and transcription factor binding were identified in the promoter regions of OsPP2 genes.

**Figure 4:**
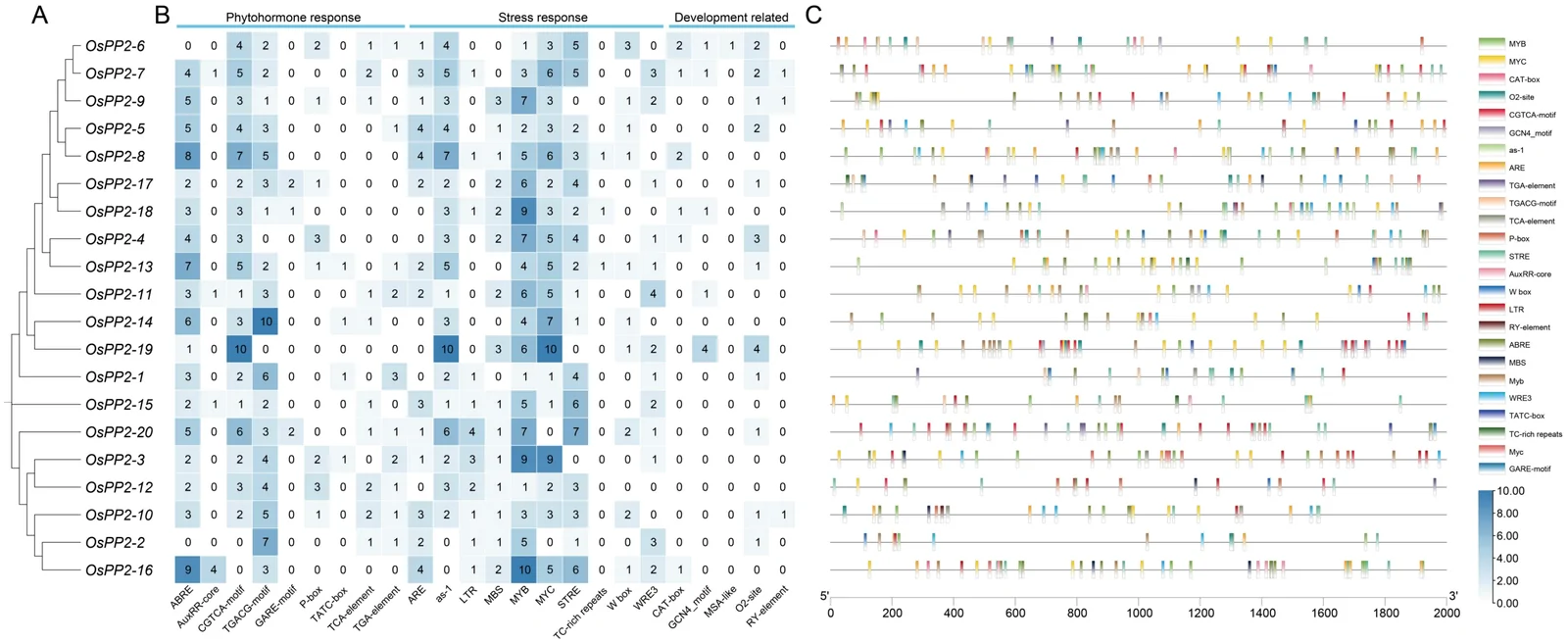
Composition and distribution of *cis*-acting elements in the promoter regions of OsPP2 genes. **(A)** Phylogenetic tree of the OsPP2 family. **(B)** Heatmap showing the abundance and distribution of predicted *cis*-acting regulatory elements in the 2-kb sequences upstream of the ATG of OsPP2 genes. **(C)** Distribution of *cis*-acting elements within the promoter regions.

Among the hormone-responsive elements, the ABA-responsive element ABRE and the jasmonic acid-responsive elements CGTCA-motif and TGACG-motif were the most abundant and were present in the promoters of most OsPP2 genes. In addition, *cis*-elements associated with auxin, gibberellin, and brassinosteroid responses were also identified. Multiple stress-responsive *cis*-elements related to drought, low-temperature stress, and defense responses were identified, including MYB, MYC, as-1, and STRE. Among these elements, MYB and MYC were particularly abundant in the promoter regions of multiple OsPP2 genes. These observations indicate that the OsPP2 promoters contain diverse predicted regulatory motifs.

### 3.6 Tissue expression profiles of OsPP2 genes

To explore the potential biological functions of OsPP2 genes in different rice tissues, publicly available transcriptomic datasets were used to analyze their expression patterns in roots, shoots, leaves, panicles, and seeds (**Figure 5**). Most OsPP2 genes exhibited distinct tissue-specific expression patterns. Among them, *OsPP2-8*, *OsPP2-10*, *OsPP2-11*, and *OsPP2–18* showed relatively higher expression in roots, whereas *OsPP2-1*, *OsPP2-3*, and *OsPP2–15* showed relatively higher expression in leaves. *OsPP2-2*, *OsPP2-7*, *OsPP2-16*, and *OsPP2–20* exhibited relatively higher expression in panicles, while *OsPP2–6* and *OsPP2–16* showed relatively higher expression in seeds. Overall, the tissue-specific expression patterns of OsPP2 genes suggest their potential involvement in rice growth and development.

**Figure 5:**
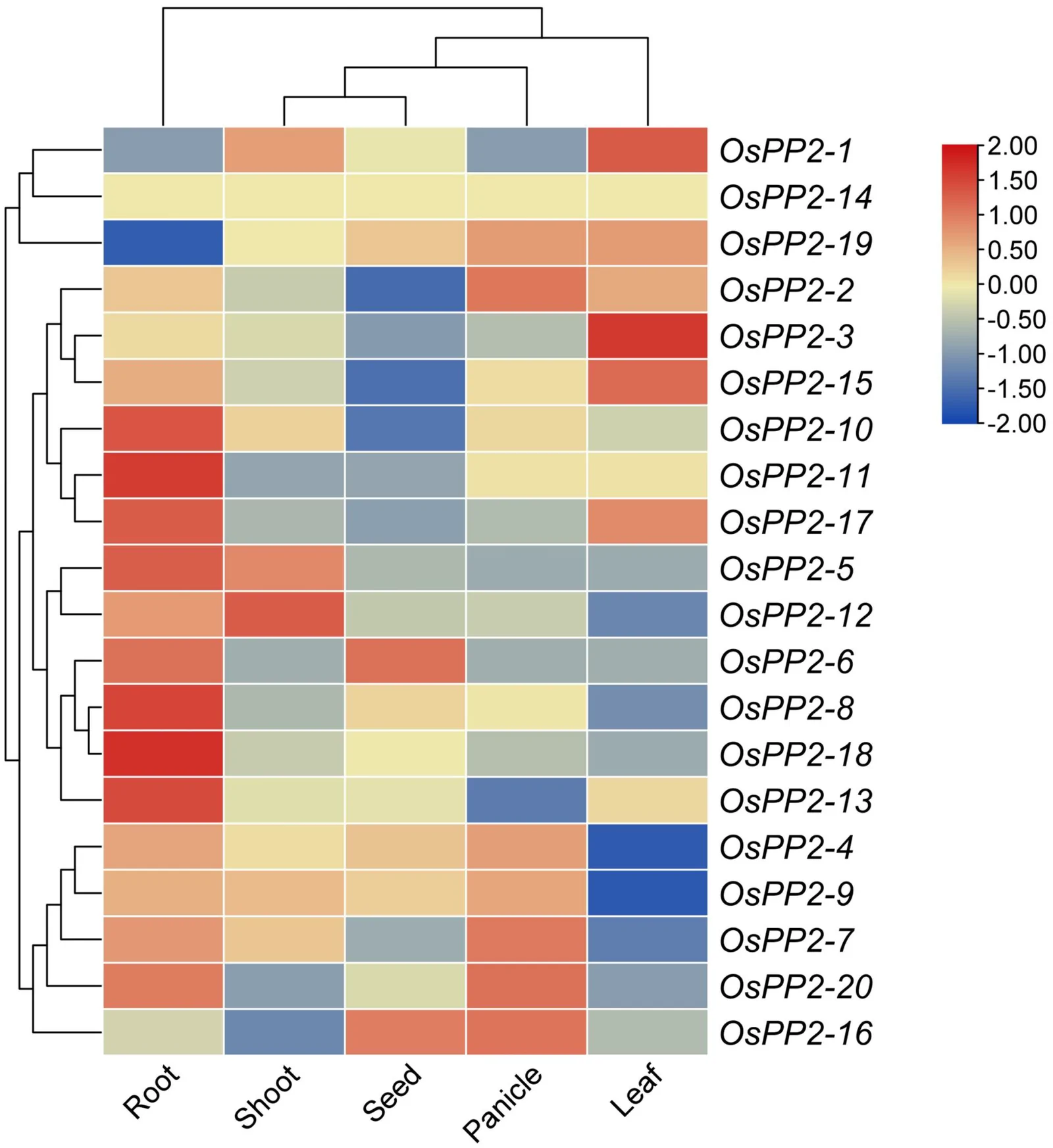
Tissue-specific expression patterns of OsPP2 genes based on publicly available transcriptomic data. Expression values were transformed using log_2_(FPKM + 1) and subsequently normalized by row-wise Z-score transformation. Red and blue indicate relatively higher and lower expression levels, respectively.

### 3.7 Expression profiles of OsPP2 genes under abiotic stresses

To investigate the potential roles of OsPP2 genes in abiotic stress responses, their expression profiles under salt, drought, heat, and cold stresses were analyzed using publicly available transcriptomic datasets. Most OsPP2 genes exhibited dynamic and stress-specific expression patterns, indicating their potential involvement in stress responses. Under salt and drought stresses, several genes, including *OsPP2-1*, *OsPP2-10*, *OsPP2-12*, *OsPP2–13* and *OsPP2–15* showed relatively higher transcript abundance than several other family members (**Figures 6A, B**). Under heat treatment, *OsPP2-2*, *OsPP2-3*, *OsPP2-8*, and *OsPP2–18* exhibited relatively higher expression levels, whereas *OsPP2-3*, *OsPP2-7*, *OsPP2-10*, *OsPP2-13*, and *OsPP2–20* showed relatively higher expression under cold treatment (**Figures 6C, D**). Notably, several OsPP2 genes responded to multiple abiotic stresses, suggesting that they may participate in responses to different environmental stresses.

**Figure 6:**
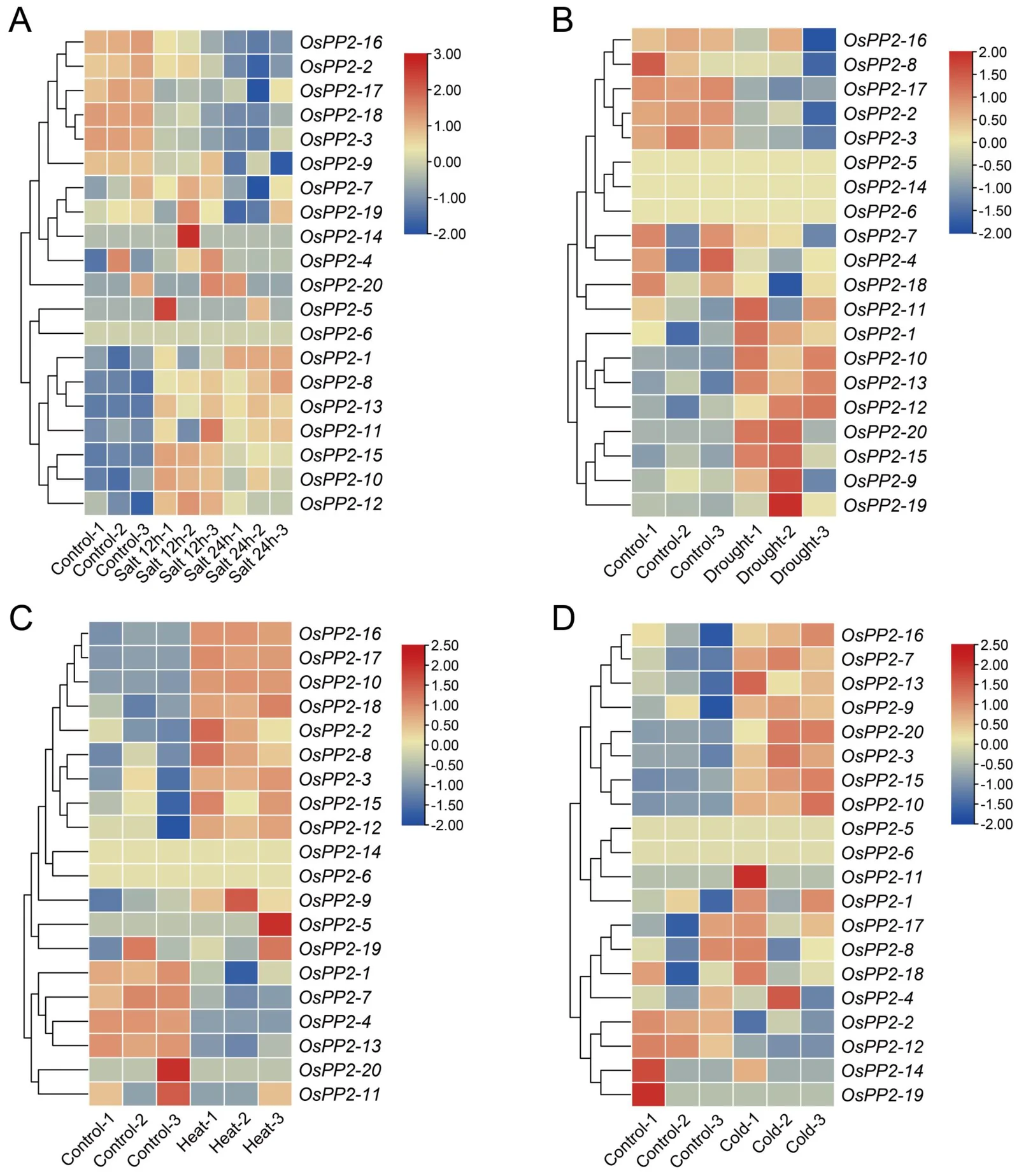
Relative expression patterns of OsPP2 genes under abiotic stresses. **(A)** Salt stress; **(B)** drought stress; **(C)** heat stress; and **(D)** cold stress. “Control1–3”, “Salt1–3”, “Drought1–3”, “Heat1–3”, and “Cold1–3” represent three biological replicates for the corresponding treatment conditions. Normalized expression values are shown as heatmaps, with red and blue indicating relatively high and low expression levels, respectively.

### 3.8 Validation of transcriptome data by qRT-PCR

To validate the transcriptome data, four representative genes (*OsPP2-1*, *OsPP2-10*, *OsPP2-13*, and *OsPP2-15*) were selected for qRT-PCR analysis under salt and PEG6000-induced osmotic stress conditions. Their expression was measured at 0, 3, 6, 12, and 24 h after stress treatment.

Under salt stress, all four genes were significantly upregulated compared with the control (**Figure 7A**). *OsPP2–10* and *OsPP2–13* exhibited the strongest transcriptional responses. Their transcript levels increased progressively, reached a peak at 12 h, and declined thereafter. *OsPP2–1* showed rapid induction at 3 h and maintained relatively high expression levels throughout the treatment period. *OsPP2–15* was markedly induced, with the highest expression observed at 12 h. Under PEG6000-induced osmotic stress, all four genes also displayed pronounced transcriptional responses (**Figure 7B**). *OsPP2–1* and *OsPP2–13* were rapidly induced at 3 h, exhibiting approximately 8-fold and 14-fold increases in transcript abundance, respectively. *OsPP2–10* exhibited sustained induction throughout the treatment period and reached its highest expression level at 12 h. In contrast, the expression of *OsPP2–15* increased gradually and reached its maximum level at 24 h.

**Figure 7:**
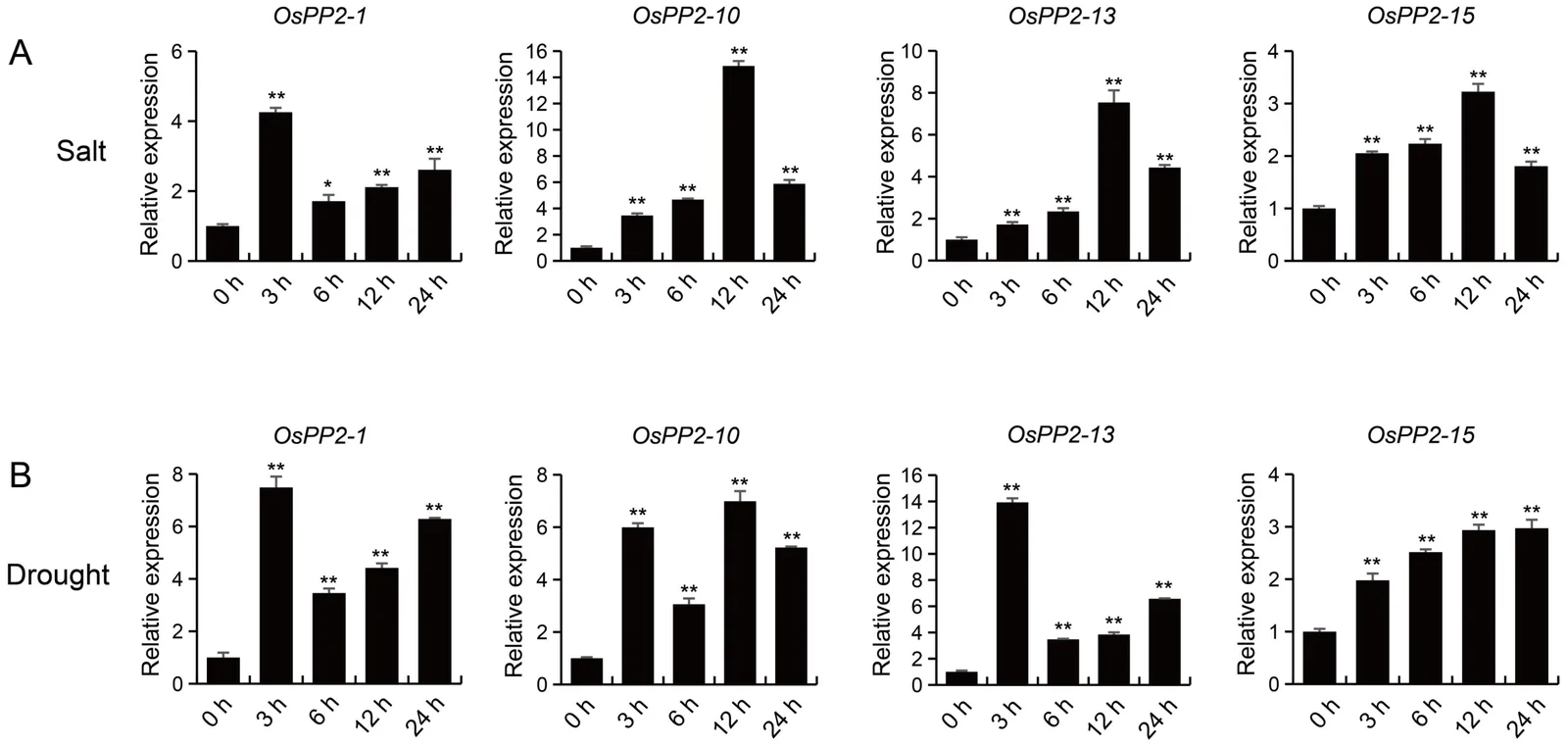
qRT-PCR validation of selected OsPP2 genes under salt and PEG6000-induced osmotic stress treatments. **(A)** Relative transcript levels of selected OsPP2 genes under salt stress. **(B)** Relative transcript levels of selected OsPP2 genes under PEG6000-induced osmotic stress. Data are presented as mean ± SD of three biological replicates. Asterisks indicate significant differences relative to the 0 h baseline (*P < 0.05; **P < 0.01).

Overall, the qRT-PCR expression patterns were highly consistent with the transcriptomic data, supporting the reliability of the RNA-seq results. Among the genes analyzed, *OsPP2–10* and *OsPP2–13* consistently exhibited stronger transcriptional responses to both salt and PEG6000-induced osmotic stress, supporting their selection as candidate stress-responsive genes for further functional studies.

### 3.9 *OsPP2–10* and *OsPP2–13* enhance salt and osmotic tolerance in yeast

To evaluate the potential roles of *OsPP2–10* and *OsPP2–13* in abiotic stress tolerance, yeast spot assays were performed under salt and osmotic stress conditions (**Figure 8**). Under control conditions, no obvious differences in growth were observed among yeast cells carrying the empty vector, pYES2-*OsPP2-10*, or pYES2-*OsPP2-13*. Under 300 mM NaCl treatment, yeast cells expressing *OsPP2–10* or *OsPP2–13* exhibited enhanced growth compared with the empty-vector control. Similarly, under PEG6000 treatment, yeast cells expressing *OsPP2–10* or *OsPP2–13* also exhibited enhanced growth relative to the control. These results indicate that heterologous expression of *OsPP2–10* and *OsPP2–13* enhances yeast tolerance to salt and osmotic stress.

**Figure 8:**
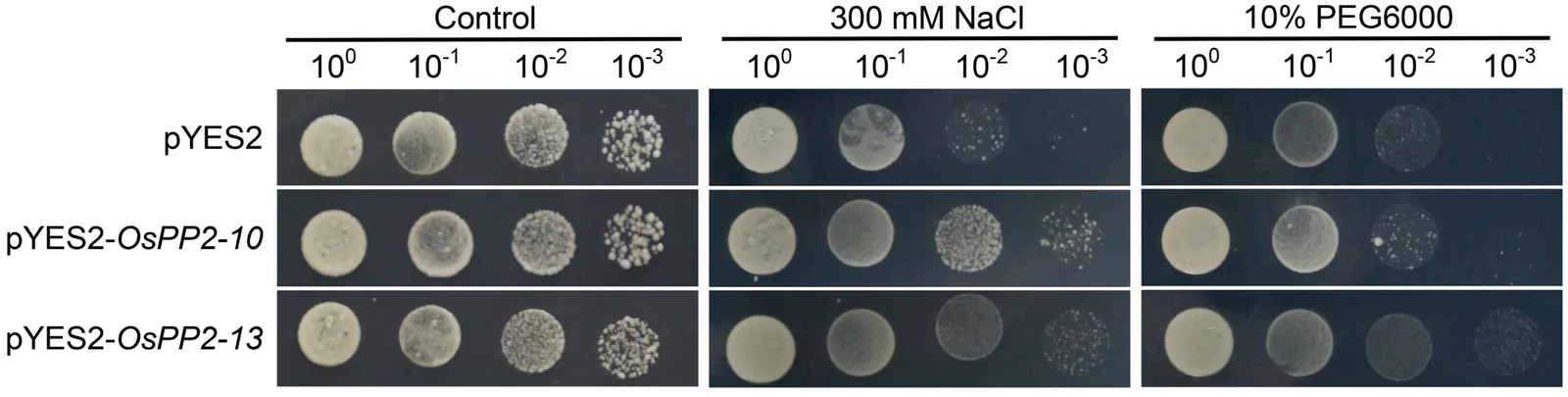
Heterologous expression of *OsPP2–10* and *OsPP2–13* enhances yeast tolerance to salt and osmotic stress.

### 3.10 Subcellular localization analysis

To further investigate the potential functions of the stress-responsive OsPP2 genes, *OsPP2–10* and *OsPP2-13*, which exhibited the strongest transcriptional induction under both salt and PEG6000-induced osmotic stresses, were selected for subcellular localization analysis. OsPP2-10–GFP and OsPP2-13–GFP fusion proteins were transiently expressed in *N. benthamiana* leaf epidermal cells via *Agrobacterium*-mediated transformation. As shown in **Figure 9**, GFP fluorescence signals from both fusion proteins were detected in the nucleus and in the peripheral regions of the cells, whereas the free GFP control was distributed throughout the entire cell. Because peripheral fluorescence in epidermal cells cannot by itself be used to unambiguously distinguish cytoplasmic from plasma membrane-associated signals, the peripheral fluorescence was conservatively described as peripheral cellular fluorescence rather than assigned to a specific subcellular compartment.

**Figure 9:**
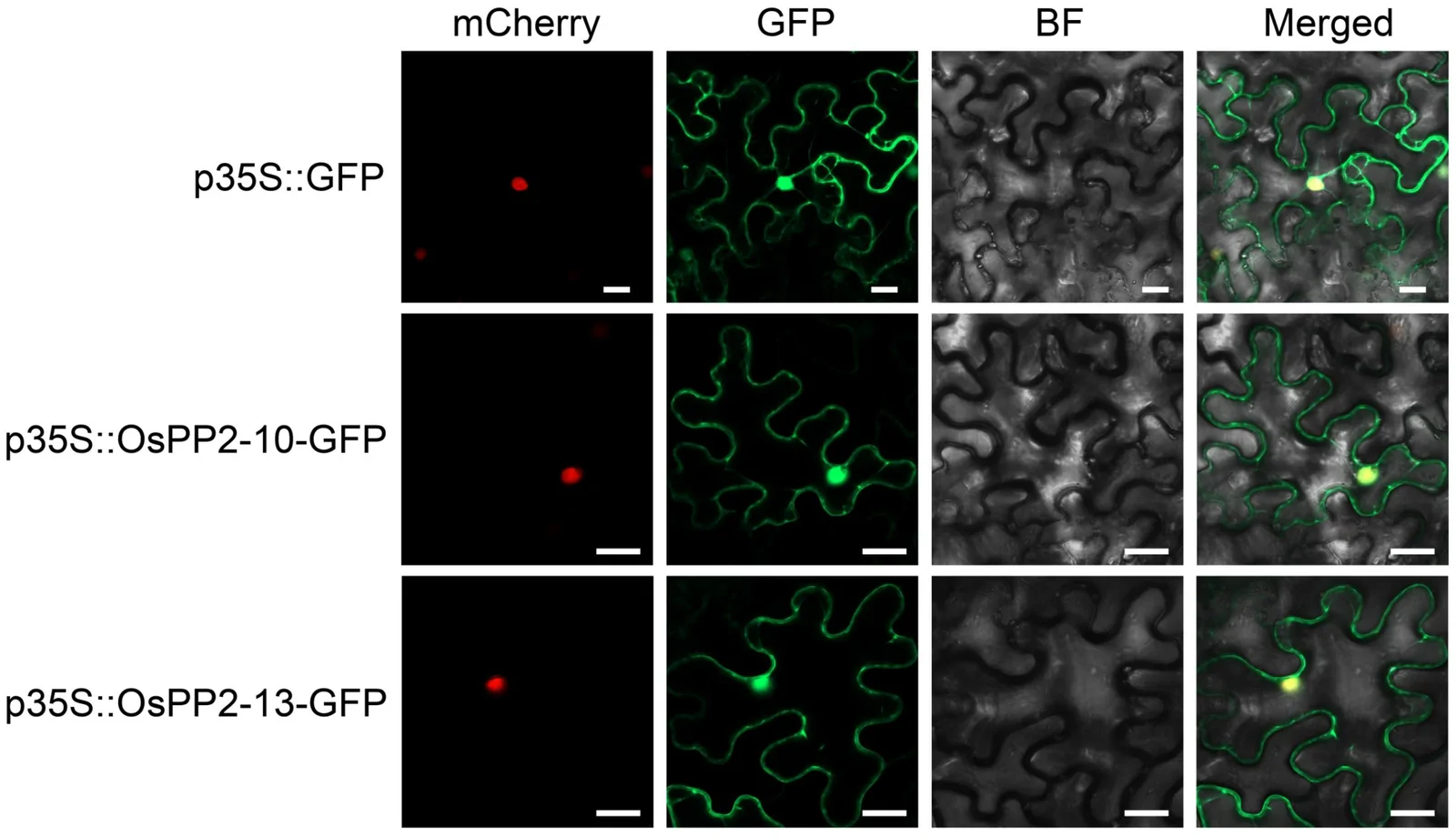
Subcellular localization of OsPP2–10 and OsPP2–13 in tobacco epidermal cells. The nuclear marker mRFP1-mCherry was specifically localized to the nucleus. Scale bar, 20 μm.

## 4 Discussion

The PP2 family has been widely implicated in phloem function, long-distance signaling, and plant responses to environmental stresses (Bobbili et al., 2023; Golecki et al., 1999; Owens et al., 2001). However, its evolutionary characteristics and biological functions in rice remain largely unclear. In this study, we performed a genome-wide identification and comprehensive characterization of the PP2 gene family in rice, revealing its evolutionary patterns, structural features, and potential involvement in abiotic stress responses. These findings provide a foundation for understanding the diversification and potential functional evolution of PP2 genes in rice.

In the present study, 20 PP2 genes were identified in the rice genome (**Supplementary Table S4**), representing a smaller family size than that reported for several dicot species (Dai et al., 2026; Dinant et al., 2003). This difference may reflect lineage-specific expansion events that occurred after species divergence. Notably, eight duplicated OsPP2 gene pairs were identified in the rice genome, including six segmentally duplicated pairs and two tandemly duplicated pairs (**Figure 3A**). These results indicate that segmental duplication made a substantial contribution to the expansion of the OsPP2 gene family, while tandem duplication also contributed to local gene expansion on chromosome 2. Ka/Ks ratios below 1 for the duplicated gene pairs were consistent with purifying selection and suggested that these genes have remained under relatively strong evolutionary constraint after duplication. Interspecific synteny analysis further revealed conserved relationships between rice PP2 genes and their counterparts in maize and sorghum, whereas fewer orthologous pairs were detected in *Arabidopsis* (**Figures 3B, C**). This pattern is consistent with the evolutionary relationships among these species and suggests that some PP2 genes have been conserved during angiosperm evolution. Interestingly, the largest number of syntenic gene pairs was observed between rice and soybean, which may be associated with the complex duplication history of the soybean genome and the subsequent retention of PP2 homologs (Schmutz et al., 2010). Collectively, these findings indicate that PP2 genes have been evolutionarily conserved during angiosperm diversification and may contribute to phloem-associated functions and plant responses to environmental stresses.

Although the OsPP2 family exhibits overall evolutionary conservation, considerable structural diversification exists among individual members. The conserved motif composition within phylogenetic groups suggests that closely related OsPP2 proteins may retain similar structural features and functional characteristics despite evolutionary divergence (**Figure 2**). In contrast, variation in exon–intron organization and motif architecture among OsPP2 genes indicates substantial structural diversification within the family. Notably, *OsPP2–20* exhibited a more complex gene structure and a distinct motif composition compared with other family members, suggesting that it may have undergone functional divergence during evolution. Structural diversification is frequently associated with changes in transcriptional regulation, protein interactions, or stress responsiveness, potentially providing a molecular basis for functional divergence among PP2 family members in rice (Bashton and Chothia, 2007; Laloum et al., 2018; Rose, 2019).

The spatiotemporal regulation of gene expression is an important determinant of gene function (Velten and Stegle, 2023). Although the OsPP2 family has undergone purifying selection, its members exhibit distinct tissue-specific expression patterns (**Figure 5**). Most OsPP2 genes displayed distinct transcript abundance patterns across tissues, suggesting that they may have undergone functional divergence and acquired distinct biological roles during plant development. Notably, *OsPP2–10* and *OsPP2–13* showed relatively higher expression in roots than in several other analyzed tissues. Given the importance of roots in water and ion uptake, this tissue-associated expression pattern may provide a basis for further investigation of their potential roles in root-related stress responses (Khan et al., 2021; Ramachandran et al., 2024). However, our qRT-PCR validation was performed using only shoot tissues, and therefore did not independently validate the root expression patterns or stress responses in roots of these genes. Further tissue-specific experiments will be required to determine whether their root-preferential expression is functionally associated with stress responses.

Promoter analysis was largely consistent with the observed expression profiles, providing additional support for our findings (**Figure 4**). Most OsPP2 promoters contained numerous stress- and hormone-responsive *cis*-regulatory elements. Among these elements, ABRE is a key *cis*-regulatory element involved in ABA signaling, whereas MYB and MYC transcription factors are widely recognized as central regulators of salt and drought stress responses (Htun et al., 2026; Singh and Roychoudhury, 2023). Consistent with these promoter features, RNA-seq data showed distinct expression patterns of multiple OsPP2 genes under salt and drought conditions, while several members also responded to cold and heat treatments (**Figure 6**). The presence of these elements suggests that the OsPP2 genes contain promoter sequences that are potentially responsive to multiple regulatory signals. Nevertheless, the presence of predicted *cis*-regulatory elements alone is insufficient to establish direct regulatory relationships. Future studies employing yeast one-hybrid assays, electrophoretic mobility shift assays (EMSA), and dual-luciferase reporter assays will be necessary to verify direct interactions between candidate transcription factors and OsPP2 promoters.

Integrated RNA-seq and qRT-PCR analyses identified *OsPP2–10* and *OsPP2–13* as promising candidates for functional characterization. Both genes showed relatively higher expression levels across several salt- and drought-related transcriptomic datasets. Their stress-responsive expression patterns were independently validated by qRT-PCR (**Figure 7**), supporting the reliability of the transcriptomic data. Notably, under salt stress, both genes reached peak transcript levels at 12 h, whereas under PEG6000-induced osmotic stress they remained induced throughout the treatment period. These patterns indicate sustained transcriptional responsiveness under the tested conditions. The induction of these genes under multiple abiotic stresses is consistent with previous reports showing that PP2 family members, including *Arabidopsis AtPP2-B11* and cucumber *CsPP2-A1*, participate in diverse abiotic stress responses (Du et al., 2022b; Jia et al., 2015; Li et al., 2023, 2014). Heterologous expression in yeast further supported the potential roles of *OsPP2–10* and *OsPP2–13* in enhancing tolerance to abiotic stress (**Figure 8**). A limitation of the present study is that the functional assays were performed in yeast rather than in transgenic or gene-edited rice. Therefore, the physiological roles of *OsPP2–10* and *OsPP2–13* in rice salt and osmotic stress tolerance remain to be experimentally determined.

The subcellular localization results provide additional insights into the potential functions of *OsPP2–10* and *OsPP2-13*. Both proteins were detected in the nucleus and in the peripheral regions of the cells (**Figure 9**), suggesting that they may participate in multiple cellular processes. Their nuclear localization raises the possibility that they may interact with nuclear proteins involved in stress-responsive regulatory pathways, whereas their peripheral cellular distribution may be associated with cytoplasmic or membrane-associated processes. This localization pattern is consistent with previous reports describing PP2 proteins as lectin-like proteins involved in stress responses (Guo et al., 2018). Together, these findings provide additional information on the cellular distribution of *OsPP2–10* and *OsPP2-13*, although their precise peripheral localization and potential roles in abiotic stress responses require further investigation.

In summary, this study presents a comprehensive characterization of the rice *PP2* gene family through integrated evolutionary, structural, and expression analyses. Multiple complementary lines of evidence, including transcriptome profiling, qRT-PCR validation, subcellular localization, and heterologous expression in yeast, consistently identified *OsPP2–10* and *OsPP2–13* as promising stress-responsive members of the OsPP2 family. However, their specific roles in rice have not yet been established. Further studies using stable overexpression and loss-of-function rice lines, combined with physiological analyses of ion accumulation, ROS status, membrane damage, water status, and antioxidant activity, will be necessary to determine their in planta functions and underlying mechanisms.

## Data Availability

The original contributions presented in the study are included in the article/**Supplementary Material**. Further inquiries can be directed to the corresponding author/s.
