# Supplementary material for: Genome-wide analysis of the rice phloem protein 2 gene family identifies OsPP2–10 and OsPP2–13 as candidate genes associated with salt and osmotic stress responses
Source: Front Plant Sci. 2026 Oct 21;17:1953442. doi: 10.3389/fpls.2026.1953442 (PMC13731405; doi:10.3389/fpls.2026.1953442)
Supplement: Supplementary file 1 [file SupplementaryFile1.docx]

**Supplementary Figure S1.** Conserved motifs of the OsPP2 gene family.

**Supplementary Table S1.** Original FPKM values used for Figure 5

| **Gene** | **Root** | **Shoot** | **Leaf** | **Panicle** | **Seed** |
| --- | --- | --- | --- | --- | --- |
| *OsPP2-1* | 0.880696 | 3.7376 | 5.79168 | 0.884084 | 2.0648 |
| *OsPP2-2* | 10.7329 | 7.99719 | 11.998 | 14.4203 | 4.87218 |
| *OsPP2-3* | 8.26738 | 5.75311 | 32.1631 | 4.19165 | 2.60553 |
| *OsPP2-4* | 2.48984 | 1.68458 | 0.055656 | 2.58742 | 2.04045 |
| *OsPP2-5* | 0.065679 | 0.052532 | 0 | 0 | 0.004297 |
| *OsPP2-6* | 0.512055 | 0 | 0 | 0 | 0.512055 |
| *OsPP2-7* | 7.682 | 5.39665 | 0.961949 | 9.72261 | 1.9247 |
| *OsPP2-8* | 3.01975 | 0.541636 | 0.241185 | 1.06257 | 1.22663 |
| *OsPP2-9* | 27.2743 | 26.014 | 8.54887 | 28.7077 | 23.8326 |
| *OsPP2-10* | 13.2924 | 5.60406 | 3.68719 | 5.42285 | 1.29312 |
| *OsPP2-11* | 4.77341 | 0.040223 | 0.960286 | 0.960286 | 0.040223 |
| *OsPP2-12* | 5.51468 | 9.43815 | 0.387667 | 1.70571 | 1.60335 |
| *OsPP2-13* | 43.1139 | 10.9087 | 14.3578 | 3.60037 | 11.2453 |
| *OsPP2-14* | 0 | 0 | 0 | 0 | 0 |
| *OsPP2-15* | 20.7815 | 17.0133 | 23.8646 | 18.762 | 13.0935 |
| *OsPP2-16* | 22.2418 | 15.5214 | 19.8428 | 38.0365 | 36.6853 |
| *OsPP2-17* | 9.99958 | 1.00485 | 6.46177 | 1.11575 | 0.588115 |
| *OsPP2-28* | 11.3636 | 1.34596 | 0.750559 | 1.09855 | 2.16787 |
| *OsPP2-19* | 0.079799 | 0.123059 | 0.141399 | 0.141399 | 0.130883 |
| *OsPP2-20* | 0.257369 | 0.059798 | 0.057721 | 0.2682 | 0.127994 |

**Supplementary Table S2.** Information on public transcriptomic datasets

| Stress | Project | Cultivar | Developmental stage | Tissue | Concentration | Duration |
| --- | --- | --- | --- | --- | --- | --- |
| Salt | PRJNA1037192 | Nipponbare | Seeding | Leaf | 150 mM NaCl | 0 h, 12h, 24h |
| Drought | PRJNA306542 | Nipponbare | 3-week old seedlings | Root | drought plot | 0 h, 24 h |
| Heat | PRJNA604026 | Nipponbare | 3-week old seedlings | aerial parts | 45℃ | 0 h, 36 h |
| Cold | PRJNA827493 | Nipponbare | Seeding | Leaf | 10℃ | 0 h, 24 h |

**Supplementary Table S3.** Original FPKM values used for Figure 6

| **Salt stress** |  |  |  |  |  |  |  |  |  |
| --- | --- | --- | --- | --- | --- | --- | --- | --- | --- |
| Gene | Control-1 | Control-2 | Control-3 | Salt 12h-1 | Salt 12h-2 | Salt 12h-3 | Salt 24h-1 | Salt 24h-2 | Salt 24h-3 |
| *OsPP2-1* | 2.379 | 1.142 | 2.506 | 6.530 | 2.426 | 4.573 | 10.077 | 10.086 | 10.109 |
| *OsPP2-2* | 72.675 | 78.743 | 107.438 | 60.491 | 68.087 | 36.477 | 16.003 | 8.866 | 18.437 |
| *OsPP2-3* | 10.325 | 10.213 | 9.649 | 5.895 | 5.621 | 4.625 | 3.939 | 3.660 | 6.287 |
| *OsPP2-4* | 0.000 | 0.227 | 0.037 | 0.082 | 0.155 | 0.220 | 0.082 | 0.077 | 0.084 |
| *OsPP2-5* | 0.000 | 0.000 | 0.000 | 0.085 | 0.000 | 0.000 | 0.000 | 0.040 | 0.000 |
| *OsPP2-6* | 0.000 | 0.000 | 0.000 | 0.000 | 0.000 | 0.000 | 0.000 | 0.000 | 0.000 |
| *OsPP2-7* | 7.157 | 8.599 | 12.670 | 10.450 | 12.887 | 11.789 | 7.312 | 4.785 | 10.523 |
| *OsPP2-8* | 0.389 | 0.347 | 0.202 | 1.413 | 1.617 | 1.892 | 1.388 | 1.982 | 2.453 |
| *OsPP2-9* | 9.676 | 9.723 | 9.612 | 7.923 | 8.124 | 9.652 | 5.908 | 8.054 | 5.343 |
| *OsPP2-10* | 19.120 | 16.564 | 27.295 | 75.527 | 67.326 | 63.085 | 34.247 | 58.088 | 37.561 |
| *OsPP2-11* | 0.000 | 0.069 | 0.000 | 0.310 | 0.000 | 0.599 | 0.248 | 0.349 | 0.382 |
| *OsPP2-12* | 29.558 | 16.288 | 9.489 | 75.120 | 112.657 | 85.456 | 45.216 | 32.840 | 33.602 |
| *OsPP2-13* | 1.906 | 1.940 | 1.639 | 96.724 | 35.470 | 96.686 | 53.783 | 101.090 | 68.313 |
| *OsPP2-14* | 0.000 | 0.000 | 0.000 | 0.000 | 0.293 | 0.000 | 0.000 | 0.000 | 0.000 |
| *OsPP2-15* | 27.653 | 29.764 | 29.270 | 78.847 | 76.167 | 68.838 | 49.325 | 53.983 | 52.317 |
| *OsPP2-16* | 24.210 | 25.731 | 30.963 | 15.020 | 11.882 | 5.888 | 3.815 | 2.940 | 4.083 |
| *OsPP2-17* | 12.122 | 13.534 | 13.000 | 8.050 | 8.572 | 7.939 | 9.157 | 5.197 | 10.694 |
| *OsPP2-18* | 4.687 | 4.432 | 4.505 | 1.461 | 1.821 | 0.813 | 0.505 | 0.475 | 1.089 |
| *OsPP2-19* | 0.201 | 0.255 | 0.273 | 0.114 | 0.399 | 0.246 | 0.000 | 0.036 | 0.314 |
| *OsPP2-20* | 0.000 | 0.000 | 0.014 | 0.000 | 0.000 | 0.017 | 0.016 | 0.000 | 0.000 |
|  |  |  |  |  |  |  |  |  |  |
| Drought stress |  |  |  |  |  |  |  |  |  |
| Gene | Control-1 | Control-2 | Control-3 | Drought-1 | Drought-2 | Drought-3 |  |  |  |
| *OsPP2-1* | 9.518 | 5.610 | 7.466 | 13.744 | 11.708 | 10.184 |  |  |  |
| *OsPP2-2* | 76.377 | 83.536 | 84.877 | 32.114 | 40.974 | 15.438 |  |  |  |
| *OsPP2-3* | 34.931 | 46.504 | 35.675 | 15.931 | 14.372 | 9.447 |  |  |  |
| *OsPP2-4* | 1.025 | 0.421 | 1.234 | 0.752 | 0.560 | 0.790 |  |  |  |
| *OsPP2-5* | 0.000 | 0.000 | 0.000 | 0.000 | 0.000 | 0.000 |  |  |  |
| *OsPP2-6* | 0.000 | 0.000 | 0.000 | 0.000 | 0.000 | 0.000 |  |  |  |
| *OsPP2-7* | 38.746 | 12.725 | 37.023 | 27.578 | 25.496 | 12.806 |  |  |  |
| *OsPP2-8* | 1.987 | 1.648 | 1.478 | 1.487 | 1.469 | 1.067 |  |  |  |
| *OsPP2-9* | 15.544 | 18.483 | 16.588 | 22.498 | 32.495 | 12.513 |  |  |  |
| *OsPP2-10* | 8.526 | 7.555 | 6.663 | 44.433 | 22.969 | 41.349 |  |  |  |
| *OsPP2-11* | 0.582 | 0.318 | 0.149 | 1.016 | 0.130 | 0.795 |  |  |  |
| *OsPP2-12* | 3.081 | 1.639 | 3.896 | 6.598 | 14.177 | 15.360 |  |  |  |
| *OsPP2-13* | 13.622 | 23.780 | 9.028 | 101.703 | 57.310 | 100.787 |  |  |  |
| *OsPP2-14* | 0.000 | 0.000 | 0.000 | 0.000 | 0.000 | 0.000 |  |  |  |
| *OsPP2-15* | 34.327 | 37.519 | 34.879 | 50.650 | 53.788 | 39.469 |  |  |  |
| *OsPP2-16* | 14.983 | 18.549 | 16.359 | 6.673 | 17.687 | 1.132 |  |  |  |
| *OsPP2-17* | 6.866 | 6.446 | 7.157 | 2.769 | 2.115 | 2.555 |  |  |  |
| *OsPP2-18* | 7.427 | 4.800 | 6.742 | 5.318 | 2.495 | 5.460 |  |  |  |
| *OsPP2-19* | 0.120 | 0.088 | 0.046 | 0.185 | 1.289 | 0.287 |  |  |  |
| *OsPP2-20* | 0.000 | 0.000 | 0.000 | 0.015 | 0.017 | 0.000 |  |  |  |
|  |  |  |  |  |  |  |  |  |  |
| Heat stress |  |  |  |  |  |  |  |  |  |
| Gene | Control-1 | Control-2 | Control-3 | Heat-1 | Heat-2 | Heat-3 |  |  |  |
| *OsPP2-1* | 2.511 | 2.349 | 2.883 | 1.289 | 0.422 | 1.511 |  |  |  |
| *OsPP2-2* | 46.318 | 38.411 | 37.134 | 62.443 | 54.756 | 48.639 |  |  |  |
| *OsPP2-3* | 29.809 | 33.187 | 28.506 | 34.617 | 34.503 | 35.341 |  |  |  |
| *OsPP2-4* | 6.540 | 6.566 | 6.126 | 1.556 | 1.497 | 1.474 |  |  |  |
| *OsPP2-5* | 0.000 | 0.000 | 0.000 | 0.000 | 0.000 | 0.044 |  |  |  |
| *OsPP2-6* | 0.000 | 0.000 | 0.000 | 0.000 | 0.000 | 0.000 |  |  |  |
| *OsPP2-7* | 16.295 | 19.473 | 18.938 | 10.662 | 8.736 | 9.520 |  |  |  |
| *OsPP2-8* | 0.548 | 0.971 | 0.567 | 1.785 | 1.477 | 1.264 |  |  |  |
| *OsPP2-9* | 16.564 | 17.847 | 18.434 | 20.124 | 22.541 | 19.563 |  |  |  |
| *OsPP2-10* | 19.814 | 19.789 | 18.976 | 60.561 | 62.449 | 61.496 |  |  |  |
| *OsPP2-11* | 0.062 | 0.000 | 0.117 | 0.000 | 0.000 | 0.063 |  |  |  |
| *OsPP2-12* | 2.857 | 2.888 | 0.000 | 6.549 | 5.500 | 6.816 |  |  |  |
| *OsPP2-13* | 14.818 | 13.487 | 14.534 | 6.035 | 5.412 | 7.494 |  |  |  |
| *OsPP2-14* | 0.000 | 0.000 | 0.000 | 0.000 | 0.000 | 0.000 |  |  |  |
| *OsPP2-15* | 24.986 | 26.043 | 22.547 | 28.579 | 26.169 | 28.111 |  |  |  |
| *OsPP2-16* | 8.296 | 11.130 | 10.868 | 78.311 | 78.293 | 67.557 |  |  |  |
| *OsPP2-17* | 2.865 | 3.065 | 3.016 | 8.833 | 7.974 | 8.168 |  |  |  |
| *OsPP2-18* | 7.482 | 5.589 | 6.395 | 11.390 | 10.997 | 12.730 |  |  |  |
| *OsPP2-19* | 0.348 | 0.546 | 0.398 | 0.432 | 0.388 | 0.551 |  |  |  |
| *OsPP2-20* | 0.000 | 0.000 | 0.030 | 0.000 | 0.000 | 0.000 |  |  |  |
|  |  |  |  |  |  |  |  |  |  |
| Cold stress |  |  |  |  |  |  |  |  |  |
| Gene | Control-1 | Control-2 | Control-3 | Cold-1 | Cold-2 | Cold-3 |  |  |  |
| *OsPP2-1* | 0.242 | 0.397 | 0.000 | 0.573 | 0.165 | 0.583 |  |  |  |
| *OsPP2-2* | 87.051 | 77.227 | 73.714 | 27.419 | 47.843 | 33.241 |  |  |  |
| *OsPP2-3* | 9.971 | 10.031 | 8.260 | 16.756 | 22.783 | 18.641 |  |  |  |
| *OsPP2-4* | 0.451 | 0.286 | 0.640 | 0.422 | 0.910 | 0.223 |  |  |  |
| *OsPP2-5* | 0.000 | 0.000 | 0.000 | 0.000 | 0.000 | 0.000 |  |  |  |
| *OsPP2-6* | 0.000 | 0.000 | 0.000 | 0.000 | 0.000 | 0.000 |  |  |  |
| *OsPP2-7* | 5.715 | 1.341 | 0.925 | 22.619 | 33.484 | 15.578 |  |  |  |
| *OsPP2-8* | 0.399 | 0.284 | 0.524 | 0.528 | 0.285 | 0.413 |  |  |  |
| *OsPP2-9* | 9.373 | 12.734 | 5.823 | 14.704 | 16.263 | 14.305 |  |  |  |
| *OsPP2-10* | 40.411 | 43.889 | 42.730 | 112.984 | 108.997 | 168.988 |  |  |  |
| *OsPP2-11* | 0.000 | 0.000 | 0.000 | 0.064 | 0.000 | 0.000 |  |  |  |
| *OsPP2-12* | 7.621 | 6.748 | 3.431 | 0.356 | 0.000 | 0.000 |  |  |  |
| *OsPP2-13* | 3.907 | 3.054 | 1.600 | 11.320 | 5.255 | 6.740 |  |  |  |
| *OsPP2-14* | 0.303 | 0.000 | 0.000 | 0.157 | 0.000 | 0.000 |  |  |  |
| *OsPP2-15* | 30.105 | 32.900 | 38.258 | 82.774 | 103.291 | 120.341 |  |  |  |
| *OsPP2-16* | 15.147 | 12.218 | 9.070 | 15.831 | 16.884 | 18.856 |  |  |  |
| *OsPP2-17* | 3.805 | 2.758 | 5.945 | 6.199 | 4.428 | 5.467 |  |  |  |
| *OsPP2-18* | 1.919 | 0.503 | 1.298 | 2.182 | 1.063 | 1.325 |  |  |  |
| *OsPP2-19* | 0.038 | 0.000 | 0.000 | 0.000 | 0.000 | 0.000 |  |  |  |
| *OsPP2-20* | 0.016 | 0.032 | 0.000 | 0.212 | 0.476 | 0.474 |  |  |  |

**Supplementary Table S4.** Identification information of OsPP2 genes

| **ID** | **AGIS ID** | **Previously reported gene name** | **Number of Amino Acid (AA)** | **Molecular Weight (Da)** | **Theoretical pI** | **Instability Index** | **Aliphatic Index** | **Grand Average of Hydropathicity** |
| --- | --- | --- | --- | --- | --- | --- | --- | --- |
| *OsPP2-1* | AGIS_Os01g005270 | — | 244 | 27560.37 | 6.36 | 38.06 | 75.53 | -0.48 |
| *OsPP2-2* | AGIS_Os02g011210 | — | 166 | 19287.3 | 9.8 | 32.68 | 72.71 | -0.406 |
| *OsPP2-3* | AGIS_Os02g040730 | — | 297 | 33310.81 | 6.21 | 39.91 | 77.21 | -0.267 |
| *OsPP2-4* | AGIS_Os02g052070 | — | 332 | 35573.49 | 5.12 | 61.02 | 78.28 | -0.108 |
| *OsPP2-5* | AGIS_Os02g052080 | — | 490 | 54278.11 | 5.22 | 49.62 | 91.22 | -0.081 |
| *OsPP2-6* | AGIS_Os02g052120 | — | 198 | 22688.11 | 6.17 | 52.13 | 82.22 | -0.163 |
| *OsPP2-7* | AGIS_Os02g052130 | — | 196 | 21700.54 | 4.99 | 58.51 | 87.24 | -0.349 |
| *OsPP2-8* | AGIS_Os02g052140 | — | 632 | 71015.75 | 5.17 | 52.98 | 81.69 | -0.342 |
| *OsPP2-9* | AGIS_Os02g052160 | — | 297 | 33431.03 | 4.9 | 63.21 | 83.16 | -0.319 |
| *OsPP2-10* | AGIS_Os03g001570 | — | 297 | 32622.36 | 7.07 | 33.54 | 83.43 | -0.121 |
| *OsPP2-11* | AGIS_Os04g018240 | — | 323 | 35345.82 | 5.73 | 45.4 | 75.82 | -0.322 |
| *OsPP2-12* | AGIS_Os04g042480 | *OsFBX148* | 304 | 33747.23 | 6.74 | 40.22 | 68.12 | -0.298 |
| *OsPP2-13* | AGIS_Os08g004370 | — | 299 | 31611.02 | 5.3 | 51.7 | 77.76 | -0.064 |
| *OsPP2-14* | AGIS_Os09g011140 | — | 207 | 22807.1 | 7.89 | 37.51 | 83.82 | -0.198 |
| *OsPP2-15* | AGIS_Os09g040060 | — | 287 | 31833.38 | 9.08 | 41.43 | 80.87 | -0.251 |
| *OsPP2-16* | AGIS_Os10g031040 | — | 310 | 34597.51 | 9.09 | 46.47 | 73.32 | -0.287 |
| *OsPP2-17* | AGIS_Os12g002520 | — | 373 | 42633.38 | 6.43 | 46.25 | 83.89 | -0.467 |
| *OsPP2-28* | AGIS_Os12g002660 | — | 302 | 34389.78 | 5.68 | 43.93 | 79.77 | -0.394 |
| *OsPP2-19* | AGIS_Os12g026850 | — | 478 | 54153.45 | 7.95 | 45.61 | 90.52 | -0.285 |
| *OsPP2-20* | AGIS_Os12g029700 | — | 1016 | 115455.8 | 9.21 | 38.61 | 83.09 | -0.38 |

**Supplementary Table S5.** Ka, Ks and Ka/Ks values of duplicated gene pairs in PP2 gene family

| Gene pair | Ka | Ks | Ka/Ks |
| --- | --- | --- | --- |
| *OsPP2-16/OsPP2-3* | 0.261581787 | 1.688568776 | 0.154913315 |
| *OsPP2-16/OsPP2-10* | 0.11869858 | 1.076313394 | 0.110282544 |
| *OsPP2-16/OsPP2-12* | 0.26819121 | 1.37752979 | 0.194689953 |
| *OsPP2-12/OsPP2-3* | 0.145891584 | 0.898209327 | 0.162424926 |
| *OsPP2-10/OsPP2-12* | 0.268756496 | 1.573190997 | 0.170835262 |
| *OsPP2-12/OsPP2-15* | 0.260557571 | 1.400552023 | 0.186039195 |
| *OsPP2-4/OsPP2-5* | 0.426953329 | 1.159911618 | 0.36809126 |
| *OsPP2-6/OsPP2-7* | 0.368078307 | 0.731070627 | 0.503478451 |

**Supplementary Table S6.** Primers used in this study

| Purpose | Primer name | Primer sequence (5′ – 3′) |
| --- | --- | --- |
| Subcellular localization | *OsPP2-10*-pRC5-CF | attaacagtctagaggtaccATGGGCGCGGGCGTGTCAGA |
|  | *OsPP2-10*-pRC5-CR | gaaccaccgccaccggatccCTTCTGAGAACTAATCACCT |
|  | *OsPP2-13*-pRC5-CF | attaacagtctagaggtaccATGGCGAGCGGCGGCGGCGG |
|  | *OsPP2-13*-pRC5-CR | gaaccaccgccaccggatccGACTACGGGCCTAAACTCCA |
| qRT-PCR | *OsACT1*-qPCR-F | TTCCTACATCGCCCTGGACT |
|  | *OsACT1*-qPCR-R | AGCCTTGGCAATCCACATCT |
|  | *OsPP2-1*-qPCR-F | GTCTATCACTTGGAGTGAAGACC |
|  | *OsPP2-1*-qPCR-R | GATAGGACCGTTCAAGCTTCC |
|  | *OsPP2-10*-qPCR-F | GATGACAGGAGATACTGGTCTC |
|  | *OsPP2-10*-qPCR-R | CTCCAACCACCTCAAACCACC |
|  | *OsPP2-13*-qPCR-F | GTCGTCGAGTGCATGGAGTT |
|  | *OsPP2-13*-qPCR-R | ACGAGATGAACTGATGGCTCC |
|  | *OsPP2-15*-qPCR-F | ATAGCTCCGAGCACATCCATG |
|  | *OsPP2-15*-qPCR-R | CGACGTGGTGATTGATCCAGA |
| Yeast heterologous expression | *OsPP2-10*-pYES2-CF | ctatagggaatattaagcttATGGGCGCGGGCGTGTCAGA |
|  | *OsPP2-10*-pYES2-CR | AcatgatgcggccctctagaTCACTTCTGAGAACTAATCA |
|  | *OsPP2-13*-pYES2-CF | CtatagggaatattaagcttATGGCGAGCGGCGGCGGCGG |
|  | *OsPP2-13*-pYES2-CR | AcatgatgcggccctctagaTCAGACTACGGGCCTAAACT |
